# Individual-Level Evaluation of the Exposure Notification Cascade in the SwissCovid Digital Proximity Tracing App: Observational Study

**DOI:** 10.2196/35653

**Published:** 2022-05-19

**Authors:** Tala Ballouz, Dominik Menges, Hélène E Aschmann, Ruedi Jung, Anja Domenghino, Jan S Fehr, Milo A Puhan, Viktor von Wyl

**Affiliations:** 1 Epidemiology, Biostatistics and Prevention Institute University of Zurich Zurich Switzerland; 2 Department of Epidemiology and Biostatistics University of California, San Francisco San Francisco, CA United States; 3 Department of Visceral and Transplantation Surgery University Hospital Zurich University of Zurich Zurich Switzerland; 4 Institute for Implementation Science in Health Care University of Zurich Zurich Switzerland

**Keywords:** digital proximity tracing, contact tracing, SwissCovid, mobile app, COVID-19, SARS-CoV-2, epidemiology, public health, tracking, surveillance, app, mHealth, evaluation, exposure, notification, observational

## Abstract

**Background:**

Digital proximity tracing (DPT) aims to complement manual contact tracing (MCT) in identifying exposed contacts and preventing further transmission of SARS-CoV-2 in the population. Although several DPT apps, including SwissCovid, have shown to have promising effects on mitigating the pandemic, several challenges have impeded them from fully achieving the desired results. A key question now relates to how the effectiveness of DPT can be improved, which requires a better understanding of factors influencing its processes.

**Objective:**

In this study, we aim to provide a detailed examination of the exposure notification (EN) cascade and to evaluate potential contextual influences for successful receipt of an EN and subsequent actions taken by cases and contacts in different exposure settings.

**Methods:**

We used data from 285 pairs of SARS-CoV-2-infected cases and their contacts within an observational cohort study of cases and contacts identified by MCT and enrolled between August 6, 2020, and January 17, 2021, in the canton of Zurich, Switzerland. We surveyed participants with electronic questionnaires. Data were summarized descriptively and stratified by exposure setting.

**Results:**

We found that only 79 (58.5%) of 135 contacts using the SwissCovid app whose corresponding cases reported to have triggered the EN also received one. Of these, 18 (22.8%) received the EN before MCT. Compared to those receiving an EN after MCT (61/79, 77.2%), we observed that a higher proportion of contacts receiving an EN before MCT were exposed in nonhousehold settings (11/18, 61.1%, vs 34/61, 55.7%) and their corresponding cases had more frequently reported mild-to-moderate symptoms (14/18, 77.8%, vs 42/61, 68.9%). Of the 18 contacts receiving an EN before MCT, 14 (77.8%) took recommended measures: 12 (66.7%) were tested for SARS-CoV-2, and 7 (38.9%) called the SwissCovid Infoline. In nonhousehold settings, the proportion of contacts taking preventive actions after receiving an EN was higher compared to same-household settings (82%, vs 67%). In addition, 1 (9%) of 11 ENs received in the nonhousehold setting before MCT led to the identification of a SARS-CoV-2-infected case by prompting the contact to get tested. This corresponds to 1 in 85 exposures of a contact to a case in a nonhousehold setting, in which both were app users and the case triggered the EN.

**Conclusions:**

Our descriptive evaluation of the DPT notification cascade provides further evidence that DPT is an important complementary tool in pandemic mitigation, especially in nonhousehold exposure settings. However, the effect of DPT apps can only be exerted if code generation processes are efficient and exposed contacts are willing to undertake preventive actions. This highlights the need to focus efforts on keeping barriers to efficient code generation as low as possible and promoting not only app adoption but also compliance with the recommended measures upon an EN.

**Trial Registration:**

International Standard Randomised Controlled Trial Number Registry 14990068; https://doi.org/10.1186/ISRCTN14990068

## Introduction

Digital proximity tracing (DPT) has been utilized by several countries as a complementary tool to enhance the effectiveness of manual contact tracing (MCT) in interrupting SARS-CoV-2 transmission chains [1-4]. Findings from population-level evaluations of the National Health Service (NHS) COVID-19 app in the United Kingdom [5] and the Corona-Warn-App in Germany [6] based on app monitoring and SARS-CoV-2 incidence data suggest that DPT exerted an important contribution to the identification of infected cases in the respective countries. Similarly, population-level data and simulations for the Swiss canton of Zurich suggest that exposure notifications (ENs) of the SwissCovid DPT app triggered voluntary quarantine recommendations in the equivalent of 5% of all contacts placed in mandatory quarantine after identification by MCT [7]. Furthermore, recent findings from the roll-out of a DPT app in Norway revealed that at least 11% of the identified contacts were exposed by a chance encounter and thus could have been missed by MCT [8]. However, despite these promising findings, early expectations regarding the role of these apps in preventing SARS-CoV-2 transmission have not been completely fulfilled [9]. This raises the key question of how the effectiveness of DPT could be improved further, which requires a better understanding of the factors influencing DPT processes.

A main determinant for DPT effectiveness is app adoption in the population [1,5]. However, many countries have struggled with relatively low uptake rates, impeding the apps from reaching their full potential [10-15]. Multiple studies have also shown differences in uptake across population subgroups relating to sociodemographic and behavioral factors, such as health and digital literacy, motivation, and trust in the government or science [12,15-18]. Yet, app adoption is not the only determinant for DPT effectiveness, and sociodemographic and behavioral factors are likely insufficient to explain further observed shortcomings along the DPT notification cascade [19-21]. For example, Salathé et al [20] found that only 2 (67%) of 3 upload authorization codes (ie, codes issued to the SARS-CoV-2-infected cases who should enter them into the app to warn their exposed contacts) were eventually uploaded [20]. Furthermore, individual-level data from an online panel comprising approximately 2000 individuals from Switzerland suggest that only 3 (75%) of 4 exposed contacts undertook the recommended actions after receiving an EN [22]. Such findings are concerning since DPT effectiveness is built on the premise that users (ie, cases after receiving the upload authorization code or contacts after receiving the EN) will undertake the necessary actions to prevent further transmission. In this context, we recently highlighted the importance of the exposure setting in prompting individuals to undertake recommended actions after receiving an EN in a study of cases and contacts identified by MCT in the canton of Zurich [23]. We found that receipt of ENs was associated with a faster time until the start of quarantine when the transmission risk occurred in nonhousehold settings, while there was no effect on time to quarantine in same-household exposure settings, where information flows are bound to be faster.

In this study, we aim to extend these previous analyses to evaluate potential contextual factors influencing the receipt of ENs and users' subsequent actions. Our analysis leverages data from confirmed case-contact pairs identified by MCT and enrolled in the Zurich SARS-CoV-2 Cohort study, which enabled us to recreate individual-level EN cascades and to study the exposure context and subsequent actions taken along the cascade. Specifically, we examine (1) the proportion of cases and contacts who fulfilled the necessary steps along the notification cascade in different exposure settings, (2) case and contact characteristics that may be associated with receipt of ENs by contacts, and (3) the type of and adherence to recommended actions among contacts who received an EN.

## Methods

### Pandemic Context

**Figure 1:**
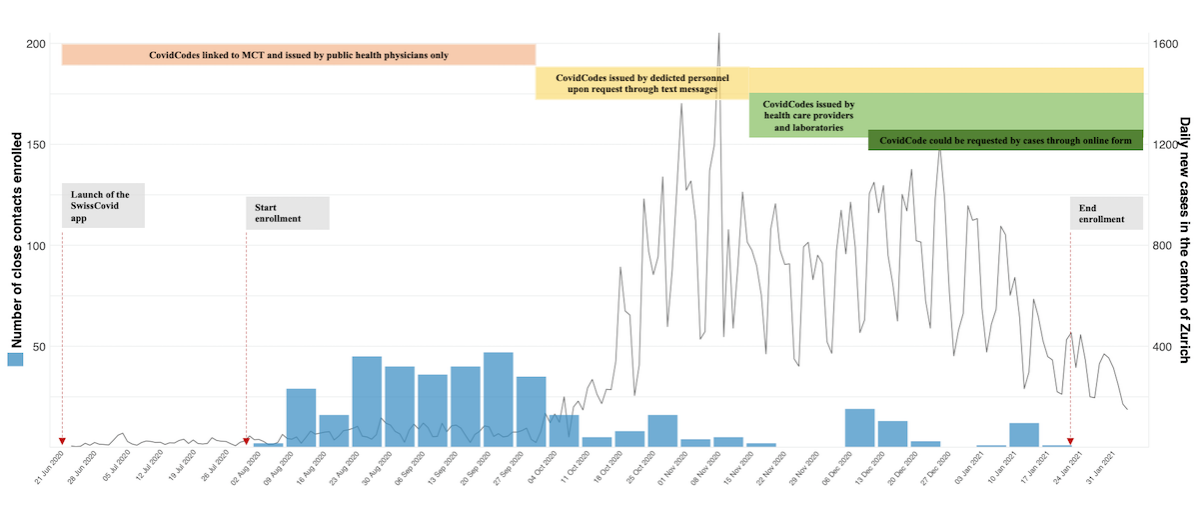
Study enrollment and events relating to key changes in processes related to DCT and MCT. DCT: digital contact tracing; MCT: manual contact tracing.

This study was conducted in Zurich, Switzerland, and analyzes data from August 6, 2020, to January 17, 2021. During the beginning of this time frame, the SARS-CoV-2 incidence in Switzerland was relatively low but steadily rising (Figure 1) [24]. At the beginning of October 2020, daily incidence sharply increased and MCT, as well as other services, such as SARS-CoV-2 testing, quickly reached capacity limits. Although relatively swift measures were undertaken to analyze and mitigate bottlenecks, their effects on reducing case numbers only materialized at the end of November 2020.

### The SwissCovid Digital Proximity Tracing App

Switzerland was among the first to launch a DPT app in June 2020 to support MCT in reducing the spread of the virus. The SwissCovid app is based on a privacy-preserving design and uses a notification cascade involving multiple sequential steps and actions taken by infected cases and their proximity contacts [2,7,21]. Upon receipt of a positive SARS-CoV-2 test, app users can request an upload authorization code (CovidCode) and enter it in the app. This triggers an EN to contacts who were within a proximity radius of less than 1.5 m for at least 15 min during the time of infectivity of the case. Therefore, an uninterrupted information flow along the notification cascade requires 3 conditions to be fulfilled: (1) cases and contacts need to be app users, (2) cases must have received and uploaded the code to trigger an EN, and (3) contacts must receive the EN. Furthermore, DPT only has an effect on preventing transmission if exposed contacts are willing to undertake the recommended preventive actions after receiving the EN. Notified contacts are thus strongly encouraged to call the SwissCovid Infoline (or, since December 2020, to complete a web form) and to get tested and enter self-quarantine, if indicated. However, these measures are not mandatory and are merely recommended by the health authorities. This stands in contrast to MCT, where quarantine and testing are mandated.

### Study Design and Participants

This study is based on data from the Zurich SARS-CoV-2 Cohort study, a prospective, case-ascertained study of 1106 individuals infected with SARS-CoV-2 (cases) and 395 of their contacts. A detailed description of the study design, its inclusion criteria, and its procedures are reported elsewhere [23]. In brief, all cases and their contacts in the canton of Zurich were identified through mandatory laboratory reporting and routine MCT by the Cantonal Department of Health and invited if they were ≥18 years old, residing in the canton of Zurich, had sufficient knowledge of the German language, and were able to follow the study procedures. After identification of eligible cases and contacts, we performed a daily random sampling of both participant populations. The sampling of cases was stratified by age, whereas contacts were randomly sampled in clusters based on the corresponding case. Sampled individuals were then invited to participate in the study.

In this study, we analyzed data from known pairs of cases and contacts. An anonymized paired data set allowing the cross-linkage of cases and corresponding contacts in the study was obtained from MCT at the Department of Health. We included only pairs for which both the case and the contact were enrolled in the study and provided data for this analysis.

### Ethical Considerations

Informed consent was obtained from all participants agreeing to participate in the study. The study protocol was approved by the responsible ethics committee of the canton of Zurich (BASEC 2020-01739) and was prospectively registered on the International Standard Randomised Controlled Trial Number Registry (ISRCTN14990068) [25].

### Data Collection

Upon enrollment, both cases and contacts completed an electronic questionnaire. For cases, information collected included sociodemographics, date of SARS-CoV-2 testing, COVID-19-related symptoms, and details regarding the suspected SARS-CoV-2 transmission event. Questionnaires for contacts included sociodemographics, presence and severity of symptoms, and details regarding the relevant exposure event (ie, setting, date, and duration). Both questionnaires included questions concerning the use of SwissCovid, including the receipt and uploading of CovidCodes by cases, as well as any ENs received by contacts. Contacts were additionally followed up at the end of quarantine, and results of any SARS-CoV-2 testing during that time were recorded. All study data were collected and managed using the Research Electronic Data Capture system (REDCap, Vanderbilt University) [26,27].

### Statistical Analysis

**Figure 2:**
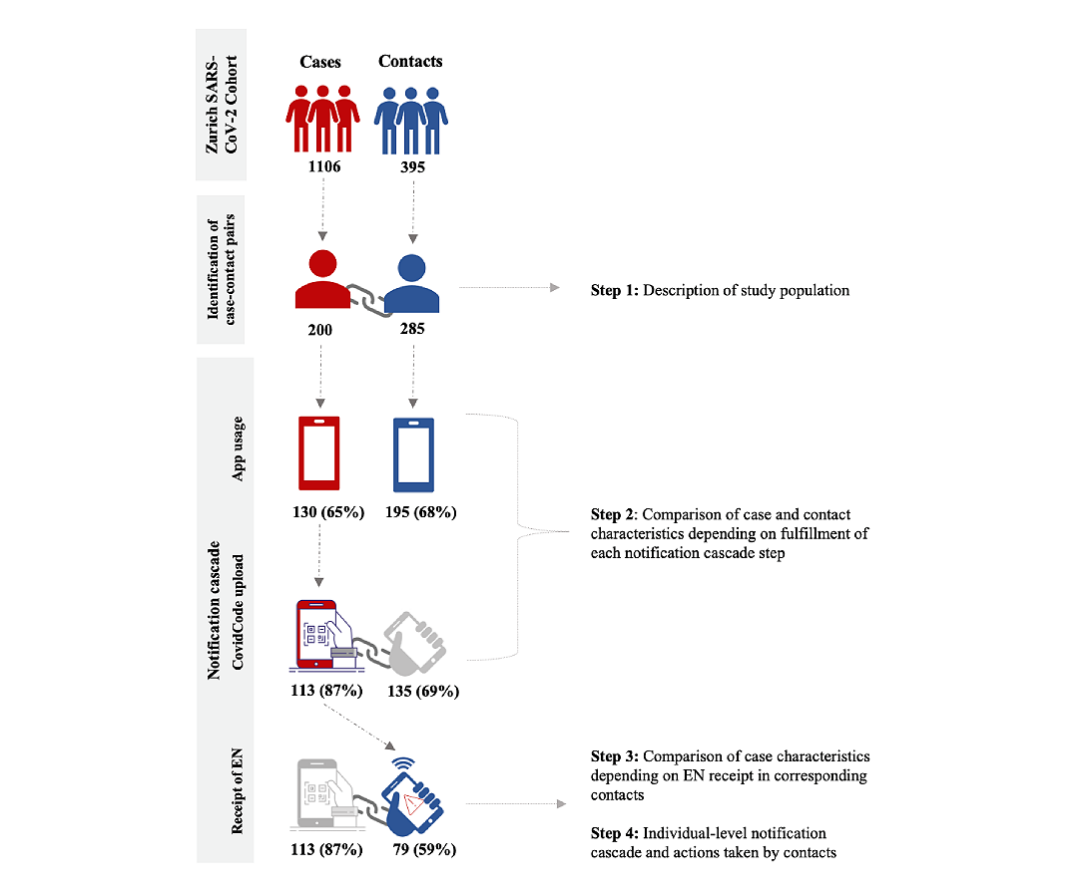
Description of the analytical steps of the study. EN: exposure notification.

The analytical steps are outlined in Figure 2. In the first step, we described the participant characteristics, including the setting in which the risk exposure occurred. In the second step, we descriptively analyzed the characteristics of cases and contacts by whether they fulfilled the necessary conditions in the notification cascade (ie, app usage among cases and contacts, cases uploading a CovidCode vs those not uploading it, and contacts receiving an EN before or after MCT vs those not receiving it). In the third step, we examined whether there were differences in the characteristics of the cases who uploaded a CovidCode and whose corresponding contacts received an EN before or after MCT. In the last step, we examined the individual-level notification cascade and the preventive actions taken by the contacts after receipt of the EN (ie, uploading the CovidCode, calling the SwissCovid Infoline, entering quarantine, or undergoing SARS-CoV-2 testing).

We presented the results for the study population overall and stratified by exposure setting as reported by the contact (ie, same-household, nonhousehold, and unknown settings). We additionally reviewed the contacts' free-text responses regarding their steps taken after receiving the EN. Responses were thematically coded and descriptively analyzed based on their context. We reported continuous variables as medians with IQRs and categorical variables as frequencies and percentages. All analyses were performed using R version 4.0.3 (R Core Team) [28].

## Results

### Description of Cases and Contacts

We identified 285 case-contact pairs within the study time frame in which both the case and the contact were enrolled in the study (n=200 cases and n=285 corresponding contacts, with a median of 1 contact per case, IQR 1-2, maximum 8). Analysis was limited to these case-contact pairs. Details of the full enrollment process of cases and contacts in the study are provided in Multimedia Appendix 1.

The median age of cases and contacts was 41 and 43 years, respectively (Table 1). Of 200 cases, 91 (45.5%) and of 285 contacts, 146 (51.2%) were female. Both populations were similar with respect to education level, employment status, Swiss nationality, and the presence of at least 1 medical comorbidity. Within case-contact pairs, the exposure occurred within the same household in 113 (39.6%) pairs and in a nonhousehold setting in 162 (56.8%) pairs, and the setting was unknown to the contacts in 8 (2.8%) pairs.

**Table 1:** Baseline characteristics of cases and contacts from 285 case-contact pairs in the Zurich SARS-CoV-2 Cohort study.

Characteristics		Cases (N=200)	Contacts (N=285)
Age in years, median (IQR)		41 (30-57)	43 (30-57)
**Sex, n (%)**			
	Female	91 (45.5)	146 (51.2)
	Male	109 (54.5)	139 (48.8)
**Education, n (%)**			
	Mandatory school	9 (4.5)	12 (4.2)
	Vocational training/baccalaureate	82 (41)	98 (34.4)
	Technical college or university studies	108 (54)	174 (61)
	Missing	1 (0.5)	1 (0.4)
**Employment status, n (%)**			
	Employed	151 (75.5)	217 (76.1)
	Student	13 (6.5)	28 (9.8)
	Unemployed/retired	35 (17.5)	39 (13.7)
	Missing	1 (0.5)	1 (0.4)
**Monthly household income,^a^ n (%)**			
	<CHF 6000 (<US $6060)	57 (28.5)	90 (31.6)
	CHF 6000-12,000 (US $6060-US $12,120)	86 (43)	113 (39.6)
	>CHF 12,000 (>US $12,120)	49 (24.5)	68 (23.9)
	Missing	8 (4)	14 (4.9)
Number of household members, median (IQR)		2 (1-3)	2 (1-3)
Missing data on household members, n (%)		4 (2)	3 (1)
**Nationality, n (%)**			
	Swiss	173 (86.5)	255 (89.5)
	Non-Swiss	27 (13.5)	30 (10.5)
**Chronic medical conditions, n (%)**			
	At least 1 self-reported comorbid condition	45 (22.5)	60 (21.1)
	Missing	3 (1.5)	7 (2.5)
**Presence of COVID-19 related symptoms, n (%)**		171 (85.5)	46 (16.1)
	Missing	0	23 (8.1)
**COVID-19 symptom severity, n (%)**			
	Asymptomatic	29 (14.5)	N/A^b^
	Mild to moderate	138 (69)	N/A
	Severe to very severe	32 (16)	N/A
	Missing	1 (0.5)	N/A
Hospitalized due to COVID-19, n (%)		2 (1)	N/A
Same-household exposure setting, n (%)		15 (7.5)	113 (39.6)
**Nonhousehold exposure setting, n (%)**			
	Private setting^c^	32 (16)	78 (27.4)
	Workplace	16 (8)	33 (11.6)
	Public space^d^	27 (13.5)	41 (14.4)
	Health care facility	1 (0.5)	0
	School/university	1 (0.5)	6 (2.1)
	Other	2 (1)	4 (1.4)
	Unknown setting	105 (52.5)	8 (2.8)
	Missing	1 (0.5)	2 (0.7)
**Country in which the exposure occurred, n (%)**			
	Switzerland	87 (43.5)	268 (94)
	Abroad	8 (4)	4 (1.4)
	Unknown	105 (52.5)	8 (2.8)
	Missing	0	5 (1.8)
**SwissCovid app use, n (%)**			
	App nonuser	69 (34.5)	88 (30.9)
	App user	130 (65)	195 (68.4)
	Missing	1 (0.5)	2 (0.7)

^a^A currency exchange rate of CHF 1 = US $1.01 was applied.

^b^N/A: not applicable (information relating to symptom severity and hospitalization related to COVID-19 only collected for cases).

^c^Settings such as friends’ apartments, private vehicles, private gatherings, or events.

^d^Settings such as restaurants, bars, shops, concerts, public transport, or religious gatherings.

### Comparison of Case and Contact Characteristics Depending on Fulfillment of Each Notification Cascade Step

Overall, 130 (65%) of 200 cases and 195 (68.4%) of 285 contacts were app users. Both cases and contacts who were app nonusers were, on average, older, and a lower proportion had a technical college or university degree, were employed, and were Swiss nationals compared to app users (Multimedia Appendix 2). There were no relevant differences between cases and contacts who used the app and those who did not in terms of their respective exposure setting, relation to the case, or country of exposure (Table 2).

Of the 130 cases who were app users, 122 (93.8%) received a CovidCode, of which 113 (92.6%) uploaded the code into the app (Table 2 and Multimedia Appendix 3). A comparison between cases uploading the code and those not uploading the code was hindered by the low number of cases not uploading the code (n=8, 6.6%). However, no relevant differences between the 2 groups were observed (Multimedia Appendix 2).

The 113 cases uploading the code were linked to 135 (69.2%) of 195 contacts using the app (Table 2). Within these 135 case-contact pairs, 79 (58.5%) of contacts received an EN through the app. Of these, 18 (22.8%) received an EN before and 61 (77.2%) after MCT. Contacts receiving an EN before MCT were more frequently exposed through nonhousehold or unknown settings compared to those receiving an EN after MCT (12/18, 66.7%, vs 34/61, 55.7%). Furthermore, the proportion of contacts whose corresponding case was a family member or a partner was lower among those receiving an EN before MCT compared to those receiving an EN after MCT (8/18, 44.4%, vs 34/61, 55.7%). Those receiving the EN before MCT were also older, on average; more frequently male (12/18, 66.7%, vs 29/61, 47.5%), and more frequently unemployed or retired (6/18, 33%, vs 4/61, 6.6%) compared to those receiving the EN after MCT (Multimedia Appendix 3). The 52 (18.2%) of 285 contacts who did not receive an EN were more often exposed in their workplace (n=11, 21.1%, vs n=1, 5.6%, receiving an EN before MCT and n=3, 4.9%, receiving an EN after MCT) and non-Swiss nationals (7/52, 13.5%, vs 0/18, 0%, and 4/61, 6.6%, respectively). We found similar results when analyzing data from all contacts (ie, not restricted to only those whose exposure case reported uploading the code); see Multimedia Appendices 4 and 5.

**Table 2:** COVID-19-related characteristics of cases (N=200) and contacts (N=285) for key steps along the notification cascade.

Characteristics		Cases^a^					Contacts^a^				
		App nonuser (N=69)	App user (N=130)	Code not uploaded (N=8)	Code uploaded (N=113)	App nonuser (N=88)		App user (N=195)	EN^b^ before MCT^c^ (N=18)	EN after MCT (N=61)	No EN (N=52)
Household exposure setting, n (%)		8 (11.6)	7 (5.4)	0	5 (4.4)	41 (46.6)		71 (36.4)	6 (33.3)	27 (44.3)	15 (28.8)
Nonhousehold exposure setting, n (%)		27 (39.1)	51 (39.2)	3 (37.5)	46 (40.7)	45 (51.1)		117 (60)	11 (61.1)	34 (55.7)	37 (71.2)
	Private setting^d^	9 (13)	22 (16.9)	2 (25)	20 (17.7)	26 (29.5)		52 (26.7)	4 (22.2)	16 (26.2)	13 (25)
	Workplace	6 (8.6)	10 (7.7)	1 (12.5)	9 (8)	10 (11.4)		23 (11.8)	1 (5.6)	3 (4.9)	11 (21.2)
	Public space^e^	9 (13)	18 (13.9)	0	17 (15)	6 (6.8)		35 (17.9)	4 (22.2)	14 (23)	10 (19.2)
	School/university	1 (1.5)	0	0	0	1 (1.1)		5 (2.6)	2 (11.1)	1 (1.6)	2 (3.9)
	Health care facility	1 (1.5)	0	0	0	0		0	0	0	0
	Other	1 (1.5)	1 (0.7)	0	0	2 (2.3)		2 (1)	0	0	1 (1.9)
	Unknown setting	34 (49.3)	71 (54.7)	4 (50)	62 (54.9)	2 (2.3)		6 (3.1)	1 (5.6)	0	0
	Missing	0	1 (0.7)	1 (12.5)	0	0		1 (0.5)	0	0	0
**Country in which the exposure occurred, n (%)**											
	Switzerland	32 (46.4)	54 (41.5)	4 (50)	46 (40.7)	86 (97.7)		182 (93.3)	17 (94.4)	60 (98.4)	51 (98.1)
	Abroad	3 (4.3)	5 (3.9)	0	5 (4.4)	0		4 (2.1)	0	1 (1.6)	1 (1.9)
	Unknown country	34 (49.3)	71 (54.6)	4 (50)	62 (54.9)	2 (2.3)		7 (3.6)	1 (5.6)	0	0
	Missing	0	0	0	0	0		2 (1)	0	0	0
**Relation of participant with SARS-CoV-2 infected individual, n (%)**											
	Family/partner	8 (11.6)	10 (7.7)	1 (12.5)	7 (6.2)	48 (54.5)		90 (46.1)	8 (44.4)	34 (55.8)	19 (36.6)
	Friend/acquaintance	14 (20.3)	22 (16.9)	1 (12.5)	19 (16.8)	20 (22.7)		59 (30.3)	6 (33.3)	17 (27.9)	18 (34.6)
	Coworker	3 (4.3)	8 (6.2)	1 (12.5)	7 (6.2)	10 (11.4)		29 (14.9)	2 (11.1)	6 (9.8)	11 (21.2)
	Customer/business partner	2 (2.9)	2 (1.5)	0	2 (1.8)	2 (2.3)		2 (1)	0	0	2 (3.8)
	Patient	1 (1.5)	0	0	0	0		0	0	0	0
	Other	7 (10.1)	15 (11.5)	0	15 (13.3)	6 (6.8)		7 (3.6)	1 (5.6)	3 (4.9)	2 (3.8)
	Case unknown	34 (49.3)	71 (54.6)	4 (50)	62 (54.8)	2 (2.3)		6 (3.1)	1 (5.6)	0	0
	Missing	0	2	1 (12.5)	1 (0.9)	0		2 (1)	0	1 (1.6)	0
**COVID-19 related symptoms and self-reported severity,^f^ n (%)**											
	Asymptomatic	9 (13)	19 (14.6)	0	16 (14.1)	N/A^g^		N/A	N/A	N/A	N/A
	Mild to moderate	47 (68.1)	91 (70)	6 (75)	82 (72.6)	N/A		N/A	N/A	N/A	N/A
	Severe to very severe	12 (17.4)	20 (15.4)	2 (25)	15 (13.3)	N/A		N/A	N/A	N/A	N/A
	Missing	1 (1.5)	0	0	0	N/A		N/A	N/A	N/A	N/A
**COVID-19 related symptoms and self-reported severity of exposure case, n (%)**											
	Asymptomatic	N/A	N/A	N/A	N/A	N/A		N/A	3 (16.7)	10 (16.3)	8 (15.4)
	Mild to moderate	N/A	N/A	N/A	N/A	N/A		N/A	14 (77.8)	42 (68.9)	35 (67.3)
	Severe to very severe	N/A	N/A	N/A	N/A	N/A		N/A	1 (5.5)	9 (14.8)	9 (17.3)
	Missing	N/A	N/A	N/A	N/A	N/A		N/A	0	0	0

^a^Missing information from 1 case on app use, 1 case on code upload, 2 contacts on app use, and 4 contacts on receipt of an EN.

^b^EN: exposure notification.

^c^MCT: manual contact tracing.

^d^Settings such as friends’ apartments, private vehicles, private gatherings, or events.

^e^Settings such as restaurants, bars, shops, concerts, public transport, or religious gatherings.

^f^Information relating to COVID-19 symptom severity was only collected in case questionnaires.

^g^N/A: not applicable.

### Comparison of Case Characteristics Depending on EN Receipt by Corresponding Contacts

In a further step, we analyzed whether there were differences in the characteristics of cases, depending on whether their corresponding contacts received the notification, before or after MCT (n=135, 69.2%; Table 2 and Multimedia Appendix 6). Overall, cases corresponding to contacts who received the EN before MCT more frequently reported having mild-to-moderate symptoms (14/18, 77.8%, vs 42/61, 68.9%) compared to cases corresponding to contacts who received the EN after MCT. Meanwhile, cases corresponding to contacts who received the EN after MCT more frequently reported having been severely or very severely affected by COVID-19 compared to those corresponding to contacts who received the EN before MCT. When analyzing case characteristics across the different exposure contexts, cases corresponding to contacts receiving an EN before MCT in the same-household setting more frequently reported being asymptomatic (2/6, 33.3%, vs 5/27, 18.5%) or having severe-to-very-severe symptoms (1/6, 16.7%, vs 1/27, 3.7%) compared to those receiving an EN after MCT (Multimedia Appendix 7). We observed similar distributions of disease severity among nonhousehold case-contact pairs as well as those with unknown exposure setting. Cases corresponding to contacts not receiving an EN generally had comparable characteristics to cases corresponding to contacts who received an EN after MCT.

### Individual-Level Notification Cascade and Actions Taken by Contacts

**Figure 3:**
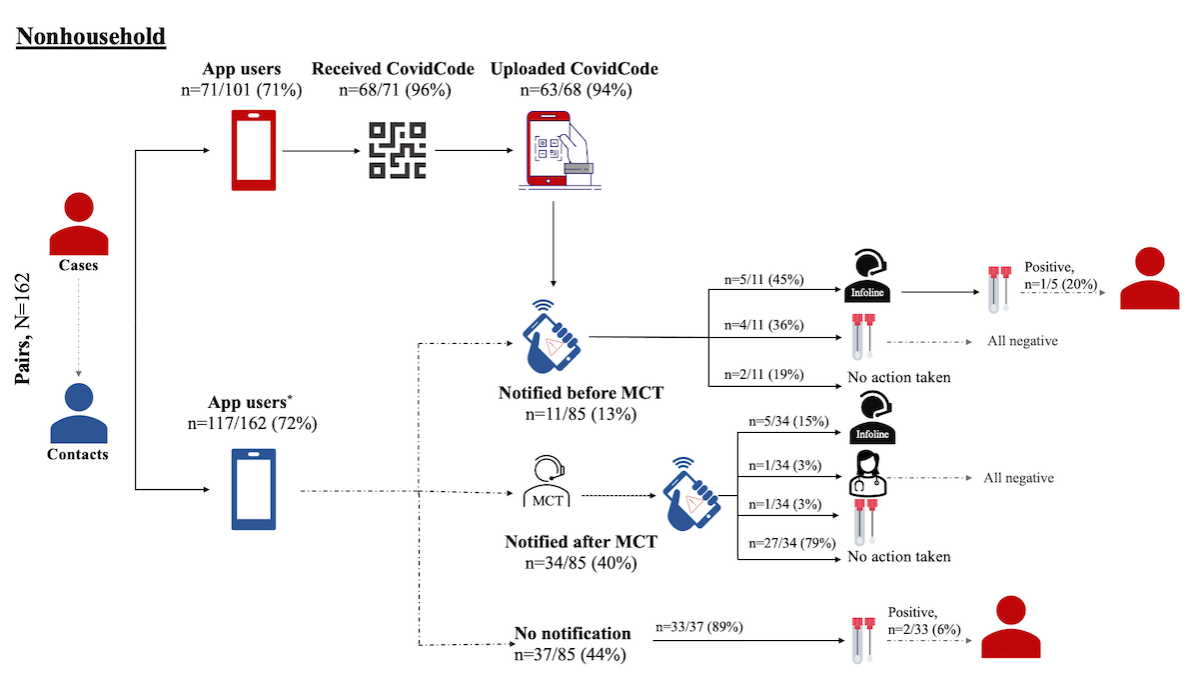
Notification cascade and preventive actions taken upon EN receipt among nonhousehold case-contact pairs (N=162, including 101, 62.3%, unique cases; *missing data on notification status in 3, 1.9%, individuals). EN: exposure notification; MCT: manual contact tracing.

**Figure 4:**
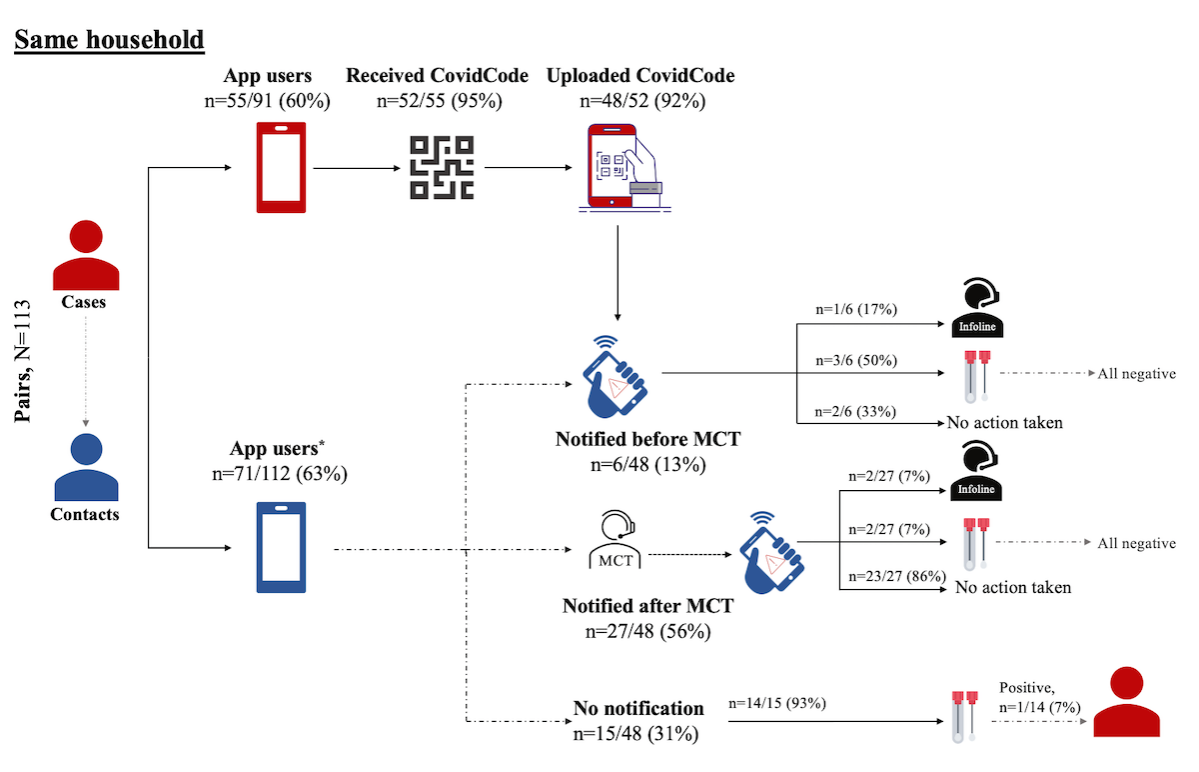
Notification cascade and recommended actions taken upon EN receipt among same-household case-contact pairs (n=113 pairs, including 91, 80.5%, unique cases; *missing data on app use in 3, 2.7%, individuals). EN: exposure notification; MCT: manual contact tracing.

Figures 3 and 4 present the sequence of events occurring along the notification cascade and the actions taken by the contacts in case-contact pairs. Figure 3 illustrates 162 case-contact pairs (n=117, 72.2%, pairs who are app users) with exposure in a nonhousehold setting, while Figure 4 shows 113 case-contact pairs (n=71, 62.8%, pairs who are app users) in a same-household setting. Multimedia Appendix 8 presents the sequence of events among the 8 pairs where the exposure setting was unknown to the contact.

In *nonhousehold case-contact pairs*, 71 (70.3%) cases and 117 (72.2%) contacts were app users. Almost all cases received a CovidCode, and 63 (94%) of 67 uploaded the received code, thereby triggering an EN. Of the 85 contacts linked to cases that uploaded the CovidCode, only 45 (53%) also received the EN, of which 11 (24%) received it before MCT. Of these 11 contacts who received the EN before MCT, 5 (45%) called the SwissCovid Infoline and a total of 9 (82%) underwent SARS-CoV-2 testing. Of these 9 individuals, 1 (11%) subsequently tested positive for SARS-CoV-2 infection. Of the 34 individuals who received an EN after MCT, the majority (n=27, 79%) did not undertake any steps as they were already in quarantine. However, 6 (18%) called the SwissCovid Infoline or responsible public health physicians and 1 (3%) reported directly seeking SARS-CoV-2 testing. Of the 37 individuals who did not receive a notification, 33 (89%) were tested for SARS-CoV-2, and 2 (6%) of them tested positive.

In *same-household case-contact pairs*, 48 (92%) of 52 cases using SwissCovid received and uploaded a CovidCode, triggering an EN in 33 (69%) of the 48 corresponding contacts using the app. Of these, 6 (18%) received the EN before MCT, and 4 (67%) reported taking recommended actions, such as undergoing testing (n=3, 75%) and calling the SwissCovid Infoline (n=1, 25%) after EN receipt. Most of those who were notified by the app after MCT (n=23, 86%) did not take any additional actions, as they were already in quarantine, and some (n=4, 17%) were also tested for SARS-CoV-2. Meanwhile, 4 (14%) undertook recommended actions after EN receipt, such as calling the SwissCovid Infoline (n=2, 50%) and seeking testing (n=2, 50%). Of those who did not receive a notification, 14 (93%) underwent SARS-CoV-2 testing, of which 1 (7%) person tested positive.

Of the 8 case-contact pairs in which the *exposure setting was unknown* to the contact, only 3 (38%) reported to be app users. Of these, 2 (67%) received and uploaded a CovidCode, triggering an EN in 1 (50%) corresponding contact, after which this contact called the SwissCovid Infoline (Multimedia Appendix 8).

## Discussion

### Principal Findings

We previously found that receipt of SwissCovid ENs was associated with earlier time to quarantine among nonhousehold contacts [23]. Here, we provide more granular data that allow a detailed assessment of the events and actions taken along the notification cascade among 285 case-contact pairs. We further interpret our results, considering the contextual changes to DPT and MCT processes over the course of the study, with the aim to provide insights that support the further optimization of current and future implementations of DPT.

The success of any DPT app strongly relies on a well-functioning EN cascade to identify and warn exposed contacts in a timely manner, as well as on the actions taken by these contacts. Our analysis suggests that a substantial proportion of ENs were not received in a timely manner and the received ENs did not always trigger the desired response in contacts. Specifically, our individual-level reconstruction of EN cascades in case-contact pairs suggests that only 79 (58.5%) of 135 exposed contacts received an EN. This finding is noteworthy because the preconditions for an EN were present in all 135 pairs (ie, both cases and contacts were app users and cases had triggered the ENs by uploading the required CovidCodes). However, we cannot exclude that some cases may have falsely reported to have uploaded the upload authorization code due to social desirability bias or that they had not yet downloaded the app at the time of the exposure and may have actually downloaded it and uploaded the code after being tested or developing symptoms. In such cases, their exposed contacts, who were identified by MCT, would not receive an EN. Furthermore, it is also possible that the risk exposure identified through MCT was not captured by the Bluetooth Low Energy signal–based technology for technical reasons (ie, proximity period too short, distance too high, diverging definition of duration or proximity by the device compared to MCT, or potential technical failures of DPT app processes) or because the devices were not carried by both individuals at the time of risk exposure. Further investigations into addressing the reasons of this gap (eg, technical improvements or education of the public on the appropriate use of the app) are required to optimize DPT performance.

Among exposed contacts who received an EN, only 18 (23%) of 79 received the EN before they were reached by MCT. These numbers should be interpreted in the light of the broader study context. The participants were enrolled in a period during which the incidence of SARS-CoV-2 was steeply rising (Figure 1). Because the issuance of CovidCodes was initially delegated to MCT personnel, the increasing workload experienced by MCT during this period also affected the timeliness of CovidCode issuance and led to cascade delays. Conversely, MCT was still relatively fast in some instances during that time (eg, if contacts were easily identifiable and contactable), thus diminishing the relative speed advantage of DPT. Our previous analysis conducted within the same cohort suggested a speed advantage of EN in nonhousehold settings but not in same-household exposure situations [23]. However, future investigations should strive to capture EN cascade steps in an even greater timely resolution. On a positive note, the majority (n=14, 77.8%) of those receiving an EN before MCT undertook recommended measures, such as seeking SARS-CoV-2 testing (n=12, 66.7%) or calling the SwissCovid Infoline (n=7, 38.9%). Similarly, another Swiss study also found that 76% of EN-notified contacts undertook a recommended action [22]. These findings stand in contrast to an experimental study from Spain, which found that only 10% of the notified contacts acted upon EN receipt by calling a designated infoline [29]. Although this low proportion could be related to the awareness of the participants about the experimental nature of that study, the inaction of contacts raised concerns about the effectiveness of the app in preventing secondary transmission. These findings emphasize the importance of having public information campaigns to increase the awareness of DPT apps. These campaigns should not only focus on highlighting the importance of using the app but also focus on adherence to the recommended actions.

We additionally examined whether case and exposure characteristics also varied noticeably by EN receipt status. Two findings, although based on limited sample sizes, may be helpful to obtain a better understanding of the notification cascade. First, contacts with workplace risk exposures seemed to be somewhat less likely to receive an EN. Only 4 (27%) of 15 individuals exposed at the workplace received a notification, as opposed to 41 (61%) of 67 for other nonhousehold exposure settings (ie, private and public spaces and schools**)**. Although coworkers may share the same workspace, the proximity or exposure time may still not reach the necessary thresholds to trigger an EN. Nevertheless, coworkers may still be identified as close contacts by MCT. Second, we found that cases whose corresponding contacts received an EN before MCT had more frequently stated having mild-to-moderate symptoms, while the cases of those receiving it after MCT more frequently mentioned severe-to-very-severe symptoms. Thus, the presence of severe symptoms could have potentially led to a delay in the uploading of CovidCodes (eg, because the case felt too ill). In those situations, allowing the possibility for proxies to swiftly trigger ENs may be considered.

In addition, we explored whether the exposure context may influence the timing of the receipt of ENs in relation to MCT as well as adherence of the contacts to recommended actions. We noted that compared to same-household settings, a higher proportion of contacts exposed in a nonhousehold setting received an EN before MCT and undertook at least 1 recommended action after receiving the EN. These actions included SARS-CoV-2 testing, with 1 (9%) of 11 received ENs having led to the identification of a SARS-CoV-2-infected case in the nonhousehold setting. Some same-household contacts also reported to have taken preventive actions after EN receipt, which may point toward a reinforcing effect of the DPT app. Current guidance on the recommended steps after receiving an EN from the SwissCovid app does not make a distinction between possible exposure settings. However, it may be worthwhile considering providing SwissCovid users with more targeted information and recommendations. For example, different recommendations could be issued for contacts knowingly exposed in household settings or through their infected partner and for contacts knowingly exposed in the nonhousehold setting or contacts with an unknown exposure context.

### Limitations

Our findings should be interpreted considering potential limitations and changes that occurred during the study period. First, study recruitment was restricted to exposed contacts identified by MCT. One advantage of DPT is to notify contacts who would otherwise be missed by MCT. This potential advantage could not be assessed within our study due to the MCT-based recruitment of study participants, thus allowing our study to only provide a partial picture of DPT effectiveness. Second, several changes to MCT and DPT processes occurred during the course of study enrollment, which may have limited the interpretation of our results (Figure 1). Upload authorization codes were initially issued by MCT personnel, and delays in receiving CovidCodes or ENs were reported during that time. This was followed by a sharp increase in case numbers in October-December 2020, during which MCT reached its capacity limits. During this period, the enrollment of contacts in the study was severely affected and had to be paused, since MCT was unable to trace an important proportion of contacts. Although a potential advantage of DPT is to compensate when MCT is overwhelmed, this setting could also not be explored due to the setup of the study. Furthermore, during that same period, several changes to CovidCode generation and MCT processes were implemented. From November 2020, the issuing of CovidCodes was improved through simplified code generation processes and by allowing laboratories and health care providers to issue the codes. Starting in December 2020, a digitally assisted form of MCT using web forms was implemented, through which cases self-reported their contacts, including contact information. At the same time, the issuance of CovidCodes was linked to the completion of the web form by the case. Although this was being implemented, the Department of Health did not have access to close contacts’ information for a certain time, which further affected our recruitment processes. In addition, we only enrolled contacts who were reached by MCT and who were still in quarantine upon first contact with our study team. In consequence, we may have missed those who were reached late or not at all by MCT and thus are most likely to have an advantage through DPT. Finally, we could not conduct any statistical analyses due to the limited sample size. However, although the findings of this study may not carry a strong statistical weight, they provide a unique observational account of the potential effects of a DPT app.

All these changes and their implications for our study may have likely led to an underestimation of the effects of SwissCovid. Conversely, some selection may have also occurred, and participants included in the study may reflect populations with higher health literacy, which may also be more likely to comply with the recommended actions and undertake preventive actions after being notified by the DPT app. Nevertheless, our results are broadly consistent with a population-based assessment of actions taken by SwissCovid app users receiving an EN in which 76% of app users took at least 1 preventive action after EN [22].

### Conclusion

In conclusion, our study provides further evidence that DPT apps can have an impact on the control of SARS-CoV-2 transmission. The detailed evaluation along each step of the DPT notification cascade within case-contact pairs demonstrates that app notifications and preventive actions taken by exposed contacts can indeed contribute to the prevention of further infections. Meanwhile, our results also show that timely compliance with the recommended measures is key for the app to exert its desired effects. It is thus important that public health messaging be targeted not just at app uptake but also for compliance with recommendations and that barriers for rapid issuance of upload authorization codes and preventive actions, such as testing and quarantine, be kept as low as possible. Further evidence collected in unknown exposure settings or times during which MCT reaches capacity limits would be desirable to judge additional contributions of DPT that could not be assessed in this study. Based on current data, DPT appears to be a relevant complementary tool in mitigating the current pandemic, while notification cascade processes and compliance are crucial determinants for its real-world effects.

## Appendix

Multimedia Appendix 1 Study enrollment and populations in the Zurich SARS-CoV-2 Cohort study.

Multimedia Appendix 2 Sociodemographic characteristics of cases and contacts for key steps along the notification cascade.

Multimedia Appendix 3 Reasons for not uploading the CovidCodes by cases who are app users and received a code.

Multimedia Appendix 4 COVID-19 characteristics of all contacts who are app users stratified by receipt of exposure notification (regardless of whether the case uploaded the code, N=195).

Multimedia Appendix 5 Sociodemographic characteristics of all contacts who are app users stratified by receipt of exposure notification (regardless of whether the case uploaded the code, N=195).

Multimedia Appendix 6 Sociodemographic characteristics of cases corresponding to contacts whose exposure case uploaded a CovidCode, stratified by notification status.

Multimedia Appendix 7 Characteristics of cases corresponding to contacts whose exposure case uploaded a CovidCode, stratified by notification status and exposure setting of the contact.

Multimedia Appendix 8 Notification cascade and preventive actions taken upon exposure notification among case-contact pairs in which the exposure setting was unknown to the exposed contact (n=8).

## Abbreviations

DPT: digital proximity tracing
EN: exposure notification
MCT: manual contact tracing
