# Supplementary material for: Individual-Level Evaluation of the Exposure Notification Cascade in the SwissCovid Digital Proximity Tracing App: Observational Study
Source: JMIR Public Health Surveill. 2022 May 19;8(5):e35653. doi: 10.2196/35653 (PMC9122110; doi:10.2196/35653)
Supplement: Multimedia Appendix 1 [file publichealth_v8i5e35653_app1.docx]

**Multimedia Appendix 1. Study enrollment and populations in the Zurich SARS-CoV-2 Cohort study**
