# Supplementary material for: Individual-Level Evaluation of the Exposure Notification Cascade in the SwissCovid Digital Proximity Tracing App: Observational Study
Source: JMIR Public Health Surveill. 2022 May 19;8(5):e35653. doi: 10.2196/35653 (PMC9122110; doi:10.2196/35653)
Supplement: Multimedia Appendix 2 [file publichealth_v8i5e35653_app2.docx]

**Multimedia Appendix 2. Sociodemographic characteristics of cases and contacts for key steps along the notification cascade**

|  | **Cases** | | | | **Contacts** | | | | |
| --- | --- | --- | --- | --- | --- | --- | --- | --- | --- |
|  | **App use** | | **Code upload by case** | | **App use** | | **Receipt of EN** | | |
|  | (among all cases, N=200)^a^ | | (among app users who received a code, N=122)^a^ | | (among all contacts, N=285)^a^ | | (among contacts who are app users and whose case uploaded a code, N=135)^a^ | | |
|  | **App non-user**, N = 69 | **App user**, N = 130 | **Code not uploaded**, N = 8 | **Code uploaded**, N = 113 | **App non-user**, N = 88 | **App user**, N = 195 | **EN before MCT**, N = 18 | **EN after MCT**, N = 61 | **No EN**, N = 52 |
| **Age, years,** Median (IQR) | 52 (35–67) | 38 (29–54) | 40 (26–59) | 37 (28–51) | 49 (30–60) | 39 (30–55) | 47 (36–58) | 35 (29–50) | 35 (29–51) |
| **Sex** |  |  |  |  |  |  |  |  |  |
| Female | 31 (45%) | 59 (45%) | 3 (38%) | 54 (48%) | 47 (53%) | 97 (50%) | 6 (33%) | 32 (52%) | 27 (52%) |
| Male | 38 (55%) | 71 (55%) | 5 (62%) | 59 (52%) | 41 (47%) | 98 (50%) | 12 (67%) | 29 (48%) | 25 (48%) |
| **Chronic comorbidity** |  |  |  |  |  |  |  |  |  |
| At least one self-reported comorbid condition | 19 (28%) | 26 (20%) | 2 (29%) | 19 (17%) | 17 (20%) | 43 (23%) | 2 (12%) | 19 (32%) | 8 (15%) |
| (Missing) | 1 | 2 | 1 | 1 | 2 | 4 | 1 | 1 | 0 |
| **Education** |  |  |  |  |  |  |  |  |  |
| Mandatory school | 8 (12%) | 1 (1%) | 0 (0%) | 1 (1%) | 4 (5%) | 8 (4%) | 0 (0%) | 2 (3%) | 2 (4%) |
| Vocational training/baccalaureate | 27 (39%) | 55 (43%) | 4 (50%) | 47 (42%) | 40 (45%) | 58 (30%) | 7 (39%) | 16 (26%) | 11 (21%) |
| Technical college or university studies | 34 (49%) | 73 (57%) | 4 (50%) | 64 (57%) | 44 (50%) | 129 (66%) | 11 (61%) | 43 (70%) | 39 (75%) |
| (Missing) | 0 | 1 | 0 | 1 | 0 | 0 | 0 | 0 | 0 |
| **Employment status** |  |  |  |  |  |  |  |  |  |
| Employed | 46 (67%) | 105 (81%) | 6 (75%) | 93 (83%) | 63 (72%) | 153 (78%) | 10 (56%) | 52 (85%) | 43 (83%) |
| Student | 5 (7%) | 8 (6%) | 1 (12%) | 7 (6%) | 9 (10%) | 19 (10%) | 2 (11%) | 5 (8%) | 5 (10%) |
| Unemployed/retired |  |  | 1 (12%) | 12 (11%) | 16 (18%) | 23 (12%) | 6 (33%) | 4 (7%) | 4 (8%) |
| (Missing) | 18 (26%) | 16 (12%) | 0 | 1 | 0 | 0 | 0 | 0 | 0 |
| **Monthly household income** | 0 | 1 |  |  |  |  |  |  |  |
| <6,000 CHF | 26 (39%) | 31 (25%) | 2 (25%) | 28 (25%) | 35 (41%) | 55 (30%) | 4 (22%) | 20 (33%) | 11 (22%) |
| 6,000-12,000 CHF | 29 (44%) | 57 (45%) | 2 (25%) | 51 (46%) | 33 (39%) | 79 (43%) | 8 (44%) | 26 (43%) | 26 (53%) |
| >12,000 CHF | 11 (17%) | 38 (30%) | 4 (50%) | 31 (28%) | 17 (20%) | 51 (28%) | 6 (33%) | 14 (23%) | 12 (24%) |
| (Missing) | 3 | 4 | 0 | 3 | 3 | 10 | 0 | 1 | 3 |
| **Number of household members,** Median (IQR) | 2 (1–2) | 2 (1–3) | 2 (1–2) | 2 (1–3) | 2 (1–3) | 2 (1–3) | 2 (1–3) | 2 (1–3) | 2 (1–3) |
| (Missing) | 1 | 3 | 0 | 3 | 1 | 1 | 0 | 0 | 0 |
| **Nationality** |  |  |  |  |  |  |  |  |  |
| Swiss | 52 (75%) | 120 (92%) | 8 (100%) | 103 (91%) | 76 (86%) | 178 (91%) | 18 (100%) | 57 (93%) | 45 (87%) |
| Non-Swiss | 17 (25%) | 10 (8%) | 0 (0%) | 10 (9%) | 12 (14%) | 17 (9%) | 0 (0%) | 4 (7%) | 7 (13%) |
| (Missing) | 0 | 0 | 0 | 0 | 0 | 0 | 0 | 0 | 0 |

^a^ Missing information from 1 case on app use, 1 case on code upload, 2 contacts on app use, and 4 contacts on receipt of EN.

CHF = Swiss Francs, EN = exposure notification, IQR = interquartile range, MCT = manual contact tracing
