# Supplementary material for: Individual-Level Evaluation of the Exposure Notification Cascade in the SwissCovid Digital Proximity Tracing App: Observational Study
Source: JMIR Public Health Surveill. 2022 May 19;8(5):e35653. doi: 10.2196/35653 (PMC9122110; doi:10.2196/35653)
Supplement: Multimedia Appendix 3 [file publichealth_v8i5e35653_app3.docx]

**Multimedia Appendix 3. Reasons for not uploading the CovidCodes by cases who are app users and received a code**

| **Reason** | **N = 8** |
| --- | --- |
| Did not work/code invalid | 2/7 (30%) |
| Received the code too late/Had already informed their contacts | 1/7 (14%) |
| Believed data has already been deleted | 1/7 (14%) |
| No contact outside their household | 1/7 (14%) |
| Did not understand what the app would trigger | 1/7 (14%) |
| No specific reason | 1/7 (14%) |
| (Missing) | 1 |
