# Supplementary material for: Individual-Level Evaluation of the Exposure Notification Cascade in the SwissCovid Digital Proximity Tracing App: Observational Study
Source: JMIR Public Health Surveill. 2022 May 19;8(5):e35653. doi: 10.2196/35653 (PMC9122110; doi:10.2196/35653)
Supplement: Multimedia Appendix 4 [file publichealth_v8i5e35653_app4.docx]

**Multimedia Appendix 4. COVID-19 characteristics of all contacts who are app users stratified by receipt of exposure notification (regardless of whether the case uploaded the code, N = 195)** ^a^

|  | **EN before MCT**, N = 18 | **EN after MCT**, N = 63 | **No EN**, N = 108 |
| --- | --- | --- | --- |
| **Exposure setting** |  |  |  |
| Household | 6 (33%) | 27 (44%) | 36 (32%) |
| Non-household | 11 (61%) | 35 (56%) | 68 (64%) |
| *Private setting^b^* | *4 (22%)* | *17 (27%)* | *29 (27%)* |
| *Workplace* | *1 (6%)* | *3 (5%)* | *19 (18%)* |
| *Public space^c^* | *4 (22%)* | *14 (23%)* | *16 (15%)* |
| *School/University* | *2 (11%)* | *1 (2%)* | *2 (2%)* |
| *Healthcare facility* | *0 (0%)* | *0 (0%)* | *0 (0%)* |
| *Other* | *0 (0%)* | *0 (0%)* | *2 (2%)* |
| Unknown setting | 1 (6%) | 0 (0%) | 4 (4%) |
| (Missing) | 0 | 1 | 0 |
| **Country in which the exposure occurred** |  |  |  |
| Switzerland | 17 (94%) | 61 (98%) | 99 (93%) |
| Abroad | 0 (0%) | 1 (2%) | 3 (3%) |
| Unknown country | 1 (6%) | 0 (0%) | 5 (5%) |
| (Missing) | 0 | 1 | 1 |
| **Relation of participant with SARS-CoV-2 infected individual** |  |  |  |
| Family or partner | 8 (44%) | 34 (55%) | 45 (42%) |
| Friend or Acquaintance | 6 (33%) | 18 (30%) | 33 (30%) |
| Coworker | 2 (11%) | 6 (10%) | 21 (19%) |
| Customer/Business partner | 0 (0%) | 0 (0%) | 2 (2%) |
| Patient | 0 (0%) | 0 (0%) | 0 (0%) |
| Other | 1 (6%) | 3 (5%) | 3 (3%) |
| Case unknown | 1 (6%) | 0 (0%) | 4 (4%) |
| (Missing) | 0 | 2 | 0 |
| **COVID-19 related symptoms and self-reported severity**^d^ |  |  |  |
| Asymptomatic | - | - | - |
| Mild to moderate | - | - | - |
| Severe to very severe | - | - | - |
| (Missing) |  |  |  |

^a^ Missing information from 1 case on app use, 1 case on code upload, 2 contacts on app use, and 6 contacts on receipt of EN

^b^ refers to settings such as friends’ apartments, private vehicles, private gatherings or events

^c^ refers to settings such as restaurants, bars, shops, concerts, public transport, religious gatherings

^d^ information relating to COVID-19 symptom severity is only collected in case questionnaires
