# Supplementary material for: Individual-Level Evaluation of the Exposure Notification Cascade in the SwissCovid Digital Proximity Tracing App: Observational Study
Source: JMIR Public Health Surveill. 2022 May 19;8(5):e35653. doi: 10.2196/35653 (PMC9122110; doi:10.2196/35653)
Supplement: Multimedia Appendix 5 [file publichealth_v8i5e35653_app5.docx]

**Multimedia Appendix 5. Sociodemographic characteristics of all contacts who are app users stratified by receipt of exposure notification (regardless of whether the case uploaded the code, N = 195)** ^a^

|  | **EN before MCT**, N = 18 | **EN after MCT**, N = 63 | **No EN**, N = 108 |
| --- | --- | --- | --- |
| **Age, years,** Median (IQR) | 47 (36–58) | 36 (29–51) | 40 (30–56) |
| **Sex** |  |  |  |
| Female | 6 (33%) | 33 (52%) | 54 (50%) |
| Male | 12 (67%) | 30 (48%) | 54 (50%) |
| **Chronic comorbidity** |  |  |  |
| At least one self-reported comorbid condition | 2 (12%) | 20 (32%) | 20 (19%) |
| (Missing) | 1 | 1 | 1 |
| **Education** |  |  |  |
| Mandatory school | 0 (0%) | 2 (3%) | 6 (6%) |
| Vocational training/baccalaureate | 7 (39%) | 17 (27%) | 33 (31%) |
| Technical college or university studies | 11 (61%) | 44 (70%) | 69 (64%) |
| (Missing) | 0 | 0 | 0 |
| **Employment status** |  |  |  |
| Employed | 10 (56%) | 53 (84%) | 86 (80%) |
| Student | 2 (11%) | 5 (8%) | 10 (9%) |
| Unemployed/retired | 6 (33%) | 5 (8%) | 12 (11%) |
| (Missing) | 0 | 0 | 0 |
| **Monthly household income** |  |  |  |
| <6,000 CHF | 4 (22%) | 20 (33%) | 28 (28%) |
| 6,000-12,000 CHF | 8 (44%) | 26 (43%) | 43 (43%) |
| >12,000 CHF | 6 (33%) | 15 (25%) | 29 (29%) |
| (Missing) | 0 | 2 | 8 |
| **Number of household members,** Median (IQR) | 2 (1–3) | 2 (1–3) | 2 (1–3) |
| (Missing) | 0 | 0 | 1 |
| **Nationality** |  |  |  |
| Swiss | 18 (100%) | 59 (94%) | 96 (89%) |
| Non-Swiss | 0 (0%) | 4 (6%) | 12 (11%) |
| (Missing) | 0 | 0 | 0 |

^a^Missing information from 6 contacts on receipt of EN

CHF = Swiss Francs, EN = exposure notification, IQR = interquartile range, MCT = manual contact tracing
