# Supplementary material for: Individual-Level Evaluation of the Exposure Notification Cascade in the SwissCovid Digital Proximity Tracing App: Observational Study
Source: JMIR Public Health Surveill. 2022 May 19;8(5):e35653. doi: 10.2196/35653 (PMC9122110; doi:10.2196/35653)
Supplement: Multimedia Appendix 6 [file publichealth_v8i5e35653_app6.docx]

**Multimedia Appendix 6. Sociodemographic characteristics of cases corresponding to contacts whose exposure case uploaded a CovidCode, stratified by notification status**

|  | **EN before MCT**, N = 18 | **EN after MCT**, N = 61 | **No EN**, N = 52 |
| --- | --- | --- | --- |
| **Age, years,** Median (IQR) | 45 (29–58) | 36 (28–47) | 33 (26–41) |
| **Sex** |  |  |  |
| Female | 11 (61%) | 25 (41%) | 26 (50%) |
| Male | 7 (39%) | 36 (59%) | 26 (50%) |
| **Level of education of case** |  |  |  |
| Mandatory school | 0 (0%) | 0 (0%) | 0 (0%) |
| Vocational training/baccalaureate | 6 (33%) | 27 (44%) | 23 (44%) |
| Technical college or university studies | 12 (67%) | 34 (56%) | 29 (56%) |
| (Missing) | 0 | 0 | 0 |
| **Employment status of case** |  |  |  |
| Employed | 14 (78%) | 48 (79%) | 41 (79%) |
| Student | 0 (0%) | 8 (13%) | 7 (13%) |
| Unemployed/retired | 4 (22%) | 5 (8%) | 4 (8%) |
| (Missing) | 0 | 0 | 0 |
