# Supplementary material for: Individual-Level Evaluation of the Exposure Notification Cascade in the SwissCovid Digital Proximity Tracing App: Observational Study
Source: JMIR Public Health Surveill. 2022 May 19;8(5):e35653. doi: 10.2196/35653 (PMC9122110; doi:10.2196/35653)
Supplement: Multimedia Appendix 7 [file publichealth_v8i5e35653_app7.docx]

**Multimedia Appendix 7. Characteristics of cases corresponding to contacts whose exposure case uploaded a CovidCode, stratified by notification status and exposure setting of the contact**

|  | **Same-household pairs** | | | **Non-household pairs**^a^ | | | **Unknown setting**^a^ | | |
| --- | --- | --- | --- | --- | --- | --- | --- | --- | --- |
|  | **EN before MCT**, N = 6 | **EN after MCT**, N = 27 | **No EN**, N = 15 | **EN before MCT**, N = 11 | **EN after MCT**, N = 34 | **No EN**, N = 37 | **EN before MCT**, N = 1 | **EN after MCT**, N = 0 | **No EN**, N = 0 |
| **COVID-19 related symptom severity of case** |  |  |  |  |  |  |  |  |  |
| Asymptomatic | 2 (33%) | 5 (18%) | 2 (13%) | 1 (9%) | 5 (15%) | 6 (16%) | 0 (0%) | 0 (0%) | 0 (0%) |
| Mild to moderate | 3 (50%) | 21 (78%) | 11 (74%) | 10 (91%) | 21 (62%) | 24 (65%) | 1 (100%) | 0 (0%) | 0 (0%) |
| Severe to very severe | 1 (17%) | 1 (4%) | 2 (13%) | 0 (0%) | 8 (23%) | 7 (19%) | 0 (0%) | 0 (0%) | 0 (0%) |
| (Missing) | 0 | 0 | 0 | 0 | 0 | 0 | 0 | 0 | 0 |
| **Level of education of case** |  |  |  |  |  |  |  |  |  |
| Mandatory school | 0 (0%) | 0 (0%) | 0 (0%) | 0 (0%) | 0 (0%) | 0 (0%) | 0 (0%) | 0 (0%) | 0 (0%) |
| Vocational training/baccalaureate | 2 (33%) | 12 (44%) | 7 (47%) | 3 (27%) | 15 (44%) | 16 (43%) | 1 (100%) | 0 (0%) | 0 (0%) |
| Technical college or university studies | 4 (67%) | 15 (56%) | 8 (53%) | 8 (73%) | 19 (56%) | 21 (57%) | 0 (0%) | 0 (0%) | 0 (0%) |
| (Missing) | 0 | 0 | 0 | 0 | 0 | 0 | 0 | 0 | 0 |
| **Employment status of case** |  |  |  |  |  |  |  |  |  |
| Employed | 5 (83%) | 23 (85%) | 11 (73%) | 8 (73%) | 25 (74%) | 30 (81%) | 1 (100%) | 0 (0%) | 0 (0%) |
| Student | 0 (0%) | 3 (11%) | 3 (20%) | 0 (0%) | 5 (15%) | 4 (11%) | 0 (0%) | 0 (0%) | 0 (0%) |
| Unemployed/retired | 1 (17%) | 1 (4%) | 1 (7%) | 3 (27%) | 4 (12%) | 3 (8%) | 0 (0%) | 0 (0%) | 0 (0%) |
| (Missing) | 0 | 0 | 0 | 0 | 0 | 0 | 0 | 0 | 0 |
| **Exposure setting of case** |  |  |  |  |  |  |  |  |  |
| Same-household | 0 (0%) | 0 (0%) | 0 (0%) | 1 (9%) | 2 (6%) | 0 (0%) | 0 (0%) | 0 (0%) | 0 (0%) |
| Non-household | 5 (83%) | 13 (49%) | 6 (40%) | 7 (64%) | 13 (38%) | 13 (35%) | 0 (0%) | 0 (0%) | 0 (0%) |
| *Private setting^b^* | *1 (17%)* | *4 (15%)* | *2 (13%)* | *4 (37%)* | *8 (23%)* | *4 (11%)* | *0 (0%)* | *0 (0%)* | *0 (0%)* |
| *Workplace* | *1 (17%)* | *4 (15%)* | *1 (7%)* | *0 (0%)* | *0 (0%)* | *2 (5%)* | *0 (0%)* | *0 (0%)* | *0 (0%)* |
| *Public space^c^* | *3 (50%)* | *5 (19%)* | *3 (20%)* | *3 (27%)* | *5 (15%)* | *7 (19%)* | *0 (0%)* | *0 (0%)* | *0 (0%)* |
| Unknown setting | 1 (17%) | 14 (51%) | 9 (60%) | 3 (27%) | 19 (56%) | 24 (65%) | 1 (100%) | 0 (0%) | 0 (0%) |
| (Missing) | 0 | 0 | 0 | 0 | 0 | 0 | 0 | 0 | 0 |
| **Relation to case** |  |  |  |  |  |  |  |  |  |
| Family or partner | 0 (0%) | 0 (0%) | 0 (0%) | 2 (18%) | 3 (9%) | 0 (0%) | 0 (0%) | 0 (0%) | 0 (0%) |
| Friend or Acquaintance | 1 (17%) | 4 (15%) | 2 (13%) | 4 (36%) | 3 (9%) | 6 (17%) | 0 (0%) | 0 (0%) | 0 (0%) |
| Coworker | 1 (17%) | 4 (15%) | 1 (7%) | 0 (0%) | 0 (0%) | 2 (6%) | 0 (0%) | 0 (0%) | 0 (0%) |
| Customer/Business partner | 0 (0%) | 0 (0%) | 0 (0%) | 0 (0%) | 1 (3%) | 0 (0%) | 0 (0%) | 0 (0%) | 0 (0%) |
| Other | 3 (49%) | 5 (18%) | 3 (20%) | 2 (18%) | 8 (23%) | 4 (11%) | 0 (0%) | 0 (0%) | 0 (0%) |
| Unknown source case | 1 (17%) | 14 (52%) | 9 (60%) | 3 (28%) | 19 (56%) | 24 (66%) | 1 (100%) | 0 (0%) | 0 (0%) |
| (Missing) | 0 | 0 | 0 | 0 | 0 | 1 | 0 | 0 | 0 |
| **Country in which case was exposed** |  |  |  |  |  |  |  |  |  |
| Switzerland | 5 (83%) | 11 (41%) | 5 (33%) | 7 (64%) | 12 (35%) | 12 (32%) | 0 (0%) | 0 (0%) | 0 (0%) |
| Abroad | 0 (0%) | 2 (7%) | 1 (7%) | 1 (9%) | 3 (9%) | 1 (3%) | 0 (0%) | 0 (0%) | 0 (0%) |
| Unknown country | 1 (17%) | 14 (52%) | 9 (60%) | 3 (27%) | 19 (56%) | 24 (65%) | 1 (100%) | 0 | 0 |
| (Missing) | 0 | 0 | 0 | 0 | 0 | 0 | 0 | 0 | 0 |

^a^ Missing information from 3 participants on notification status in non-household pairs and 1 participant in pairs with unknown exposure setting.

^b^ refers to settings such as friends’ apartments, private vehicles, private gatherings or events

^c^ refers to settings such as restaurants, bars, shops, concerts, public transport, religious gatherings
