# Supplementary material for: Individual-Level Evaluation of the Exposure Notification Cascade in the SwissCovid Digital Proximity Tracing App: Observational Study
Source: JMIR Public Health Surveill. 2022 May 19;8(5):e35653. doi: 10.2196/35653 (PMC9122110; doi:10.2196/35653)
Supplement: Multimedia Appendix 8 [file publichealth_v8i5e35653_app8.docx]

**Multimedia Appendix 8. Notification cascade and preventive actions taken upon exposure notification among case-contact pairs in which the exposure setting was unknown to the exposed contact (n=8).** ^*^missing data on notification status in 1 individual
